# Unraveling protein misfolding diseases using model systems

**DOI:** 10.4155/fso.15.41

**Published:** 2015-09-01

**Authors:** Sara Peffer, Kimberly Cope, Kevin A Morano

**Affiliations:** 1 University of Texas Graduate School of Biomedical Sciences, Houston, TX 77030, USA; 2 Department of Microbiology & Molecular Genetics, University of Texas Medical School, Houston, TX 77030, USA

## Abstract

Experimental model systems have long been used to probe the causes, consequences and mechanisms of pathology leading to human disease. Ideally, such information can be exploited to inform the development of therapeutic strategies or treatments to combat disease progression. In the case of protein misfolding diseases, a wide range of model systems have been developed to investigate different aspects of disorders including Huntington's disease, Parkinson's disease, Alzheimer's disease as well as amyotrophic lateral sclerosis. Utility of these systems broadly correlates with evolutionary complexity: small animal models such as rodents and the fruit fly are appropriate for pharmacological modeling and cognitive/behavioral assessment, the roundworm *Caenorhabditis elegans* allows analysis of tissue-specific disease features, and unicellular organisms such as the yeast *Saccharomyces cerevisiae* and the bacterium *Escherichia coli* are ideal for molecular studies. In this chapter, we highlight key advances in our understanding of protein misfolding/unfolding disease provided by model systems.

**Figure 1.**
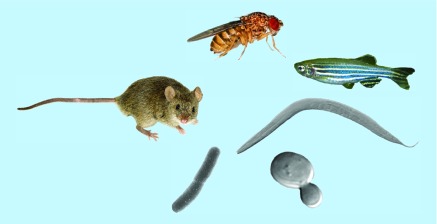
**Model systems.** Shown clockwise from left, common laboratory mouse (*Mus musculus*), fruit fly (*Drosophila melanogaster*), zebrafish (*Danio rerio*), roundworm/nematode (*Caenorhabditis elegans*), baker's yeast (*Saccharomyces cerevisiae*) and bacteria (*Escherischia coli*). Images obtained through Creative Commons license, with specific credit to the following: mouse, G. Shuklin; fly, Bbski; worm, kbradnam; bacteria, Rocky Mountain Laboratories.

**Figure 2.**
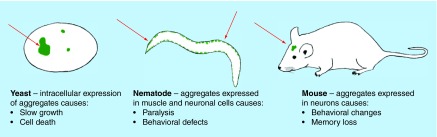
**Organism-specific phenotypes resulting from protein aggregation.** Salient phenotypes arising from protein aggregation are described. In most cases severity scales with the amount or frequency of aggregation, allowing quantifiable assessment of physiological impact.

## Animal model systems

A range of model systems are being utilized to probe the intricacies of protein misfolding diseases (Figure 1). In addition to suitability for the type of question being addressed (i.e., molecular vs physiological), a number of additional factors contribute to the choice of experimental system, such as differential costs to house, feed and maintain populations (Table 1). These considerations are amplified with genetic studies, where both generation time and time to generate knockout or transgenic lines must be taken into account.

## Rodents

Rodents are the go-to models for neurodegenerative disease. While aging **‘wild type’** rodents have not proven particularly effective (e.g., they do not spontaneously develop Aβ plaques and tau pathology associated with AD) [1], pharmacologically and genetically altered animals have offered some insight. Two drugs, scopolamine and mecamylamine [2], as well as Aβ peptides themselves [3], have been injected into rodent brains resulting in cognitive deficits similar to those observed in human AD (Figure 2). However, the full complexity of the disease in humans is not recapitulated as disease symptoms do not progress over time. Systemic injection of inhibitors of succinate dehydrogenase, such as 3-nitropropionic acid and malonate, was shown to produce lesions in the medium spiny neurons of the striatum and create the same motor impairments as those seen in Huntington's patients [4,5]. A downside of malonate, however, is that it does not readily cross the blood–brain barrier and, thus, must be injected intrastriatally which can damage neurons themselves. Parkinson's models rely on drugs that cause changes in dopamine levels or degeneration of dopaminergic neurons (e.g., 6-hydroxydopamine [6-OHDA]) [6]. Importantly, clinically relevant Parkinson's disease (PD) drugs have shown efficacy in the 6-OHDA model [7].

For AD, the majority of cases in humans occur in patients with wild-type amyloid precursor protein (APP), thus, expressing wild-type human APP in rodents would seem to be the most relevant model [8]. The first attempt involved expressing the human Aβ peptide directly under a promoter, thus bypassing the need for processing; however the Aβ did not develop into amyloid plaques [9]. Expression of the entire gene via a yeast artificial chromosome led to correct protein synthesis and alternative splicing, but only produced amyloid deposits when expressed at very high levels in aged mice [10]. Several mutations linked to familial, early-onset AD have also been expressed in rodents with moderate success. As an example, mice expressing the London APP mutation developed senile plaques, neurofibrillary tangles and diffuse Aβ deposits, resulting in synaptic loss and gliosis and problems with spatial learning and memory that increased with age despite lack of neuronal death [11,12]. For Huntington's disease (HD), models expressing human huntingtin with an expanded polyglutamine region develop a progressive syndrome akin to HD. Expressing only the N-terminal fragment of exon 1 of huntingtin leads to motor defects and weight loss similar to that seen in HD [13]. Mouse models lacking or overexpressing α-synuclein, a causative agent of PD, suffer mitochondrial damage, increase in size and number of glial cells and degeneration of motor neurons [14]. Interestingly, the wild-type α-synuclein sequence in mice is identical to one of the mutant forms in man associated with familial PD; however, loss of nigral dopaminergic neurons is not induced upon overexpression as it is in the human disease. Mice transgenic for the PD-linked LRRK2 also exhibit dopaminergic dysfunction and some behavioral deficits, but no noticeable nigral cell degeneration [15]. Therefore both pharmacological and transgenic rodent models of neurodegenerative disease manifest some, but not all of the human pathologies and may be selectively useful in understanding features of the respective diseases.

### Drosophila melanogaster

The fruit fly *Drosophila melanogaster* provides some clear advantages over higher animals including the relative ease with which the genome can be manipulated, the greatly reduced costs of maintaining fly colonies and the ability to perform high-throughput screens [1]. Transgenic flies engineered to carry human APP and β-secretase (flies lack this critical processing enzyme) exhibit deposition of Aβ plaques, age-dependent neurodegeneration, shortened lifespan and defects in wing vein development [16]. Other groups have fused the Aβ fragment to a secretion signal peptide and observed both intracellular and extracellular accumulation of the fragment which lead to neurodegeneration, problems with olfactory memory and locomotion and reduced lifespan [17–19]. For PD, a large number of human genes such as *parkin*, *UCH-L1*, *PINK1*, *DJ-*1 and *LRRK2* have highly conserved homologs in *Drosophila* [20]. Knockouts of PD-related homologs including *parkin, DJ-1* and *LRRK2* result in flies that exhibit motor deficits that can be restored following treatment with l-dopa, dopamine agonists (pergolide, bromocriptine) or muscarinic antagonists (atropine) [21].

Expressing a polyglutamine-expanded version of human huntingtin (Htt-Q128) induced progressive symptoms akin to those of Huntington's in humans: motor skills were impaired, huntingtin aggregates that disrupted axonal transport formed in the cytoplasm and neurites, and lifespan was shortened [22]. The aggregation of polyglutamine expanded proteins resulting in defective axonal transport was specific to polyglutamine expanded Htt; neither Htt lacking a polyglutamine tract, nor a standalone polyglutamine tract, nor a polyglutamine expanded version of spinocerebellar ataxia type 3 protein displayed any such phenotype. In a similar model, both genetic and pharmacological reduction of the histone deacetylases Rpd3 and Sir2 had a neuroprotective effect but did not increase longevity to wild-type levels [23]. One group found that therapeutic strategies targeting multiple pathways of aggregate formation and neural degeneration are ideal for treatment of Huntington's when they discovered two combinations of drugs that worked more effectively than any single of the drugs used alone, even at higher concentrations [24]. Another observed that peptides engineered to bind the Htt protein, dubbed intrabodies, were effective at increasing lifespan to wild-type levels [25]. Overexpression of the molecular chaperone, Hsp70, in the Drosophila model suppressed neurotoxicity but not aggregation, suggesting that it is the soluble Htt protein that is toxic [26]. Interestingly, directed expression of Hsp70 also prevented neurodegeneration in the *Drosophila* PD model, suggesting a therapeutic role for molecular chaperones in various diseases of protein misfolding and aggregation [27]. Indeed, molecular chaperones colocalize with aggregates in Alzheimer’s, Huntington’s, Parkinson’s, amyotrophic lateral sclerosis (ALS) and a few other protein misfolding diseases [28].

### Danio rerio

Zebrafish (*Danio rerio*) confer similar advantages as *Drosophila*. In AD, GSK-3b is abnormally upregulated and may be a potential therapeutic drug target [29]. Inhibition of GSK-3b in zebrafish results in a headless embryo allowing screening for potential AD therapeutic drugs. In one screen, 4000 chemical compounds were tested and one, named 3F8, was found to be a potent inhibitor. Another screen involved derivatives of benzo[ε]isoindole-1,3-dione: several showed inhibitory activity with 7,8-dimethoxy-5-methylbenzo[e]isoindole-1,3-dione being the strongest effector [30,31]. As in the mouse model, 6-OHDA and 1-methyl-4-phenyl-1,2,3,6-tetrahydropyridine (MPTP) reduce brain levels of dopamine, noradrenaline and histamine [32]. The drugs reduce the amount of swimming, but the effects are transient and the fish return to normal within 8 days. **Knockdown** of PD-related genes via addition of morpholinos at the embryonic stage of growth has produced fish with a wide variety of PD-relevant phenotypes including neurodegeneration of dopaminergic cells in the posterior tuberculum, and reduced swimming; however these symptoms are not always correlated [33,34].

### Caenorhabditis elegans

*Caenorhabditis elegans* is one of the simplest multicellular organisms available for study as a model system. This small, translucent nematode has a well-defined cell lineage, short life span, large brood size, greater genetic tractability than other animals and carries orthologous genes to more than 40% of known genes responsible for human diseases [35]. The ability to manipulate the *C. elegans* genome allows for the expression of recombinant human proteins in various cell types/tissues, compartments or the whole animal. Genetic deletions and gene knock-downs with RNA interference (RNAi) are also techniques used in exploiting *C. elegans* to study the molecular pathways requiring the protein of interest as well as to study the effects of drug therapies at the organismal, tissue and cellular levels (Figure 2).

Modeling Huntington's disease in *C. elegans* requires the expression of a recombinant fragment of Huntingtin protein (Htt) containing multiple polyglutamine (polyQ) repeats in either muscle or neuronal cells using appropriate tissue-specific promoters. In muscle, Htt induces paralysis and death, providing a ready phenotype to use in drug screens. In one drug screen, the paralytic phenotype was used to identify a novel hydroxylamine derivative NG-054 that suppressed the polyQ-associated toxicity even when administered after disease onset [36]. Another screen produced meclizine, an already approved antiemetic treatment, as a potential therapeutic for Htt toxicity based on improved touch response in worms experiencing a reduction in touch sensitivity due to neuronal polyQ expression [37].

Transgenic worms engineered to express Aβ or mutant tau protein in muscle cells and neurons have been used to investigate the molecular pathways involved in AD plaque pathology. *C. elegans* also produces APL-1, an essential protein related to human APP, which is not secreted and does not contain the Aβ peptide but is used to study APP-linked molecular pathways [38]. Transgenic Aβ expression in worms has previously uncovered a link between insulin/insulin growth factors, aging and stress response genes that is recapitulated in worm models using fragments of the worm's own APL-1 [39]. The link between insulin, aging and AD may also be relevant to the human disease. For example, the characteristic weight loss experienced by AD patients may be due to changes in metabolism. Senile plaques, as found in many tauopathies, are evident in postmortem diagnosis of AD and a small molecule screen identified the antipsychotic compound azaperone as a reducer of insoluble tau in *C. elegans* neurons [40]. Likewise, formation of plaques containing α-synuclein, neurofilament and ubiquitin, termed Lewy bodies, is a pathological hallmark of PD. *C. elegans* does not produce an ortholog to α-synuclein, however transgenic worms can be made to overexpress the human protein in various neurons to investigate the molecular pathways associated with Lewy body toxicity. One study revealed the worm protein DNJ-27, orthologous to human ERdj5, protects against α-synuclein aggregation by modulating mitochondrial integrity [41]. This finding implicates the process of endoplasmic reticulum (ER)-associated degradation, a known role for ERdj5, in resistance to Lewy body toxicity. Moreover, overexpression of DNJ-27/ERdj5 provides a broadly protective effect against neurodegeneration resulting from aggregation of α-synuclein, Aβ and polyQ proteins, indicating that there may be therapeutic targets with promise for treatment of multiple neurodegenerative diseases [41].

Studying ALS proves to be difficult due to the wide variety of causes from familial forms of the disease (fALS) to spontaneous instances. There are specific mutations commonly associated with fALS that can be recreated in *C. elegans* including mutations in superoxide dismutase 1 (SOD1), a ubiquitous enzyme responsible for detoxification of superoxide anions and mutations in TDP-43 or FUS (fused in sarcoma; mTDP-43, mFUS), two conserved nucleic acid binding proteins. When mutant, human SOD1 (mSOD1) is overexpressed in *C. elegans* motor neurons, the worms experience age-dependent paralysis as well as motor neuron degeneration mirroring that is seen in ALS patients [42]. Similarly, when mTDP-43 and mFUS are expressed in transgenic worms, the animals develop progressive motility defects that occur over a period of days on solid agar surfaces and is exaggerated to just a few hours in liquid media [43]. The rapid onset of paralysis in liquid media provides a clear phenotype to screen for therapeutics that reduce, reverse or halt symptom progression, and was used to identify the redox-active compound methylene blue as an inhibitor of ALS-associated motility defects. These results identified a novel function of mTDP-43 and mFUS in ALS progression and underscore the links between protein aggregation and cellular reactive oxygen species [43].

### Saccharomyces cerevisiae

Many fundamental cellular functions, including pathways involved in neurodegeneration such as protein folding and secretion, are highly conserved between the yeast *S. cerevisiae* and humans [44]. Genetic manipulation of this organism is trivial compared with the models previously described, and powerful genomic and proteomic tools have been developed that are unsurpassed in any other system. Despite nearly a billion years of evolutionary divergence, recent estimates that a fifth of yeast genes have human disease orthologs lends support to functional discovery investigations using this model [45]. When human α-synuclein was overexpressed, cells experienced accumulation of lipid droplets in the cytoplasm, problems with vesicular transport, ER and mitochondrial stress, dysfunction of the ubiquitin proteasome and induction of the heat shock response (summarized in [46]). A variety of mutations in human SOD1 have been linked to ALS; however, when different hSOD1 alleles are expressed in yeast, distinct outcomes are observed [47]. This supports the hypothesis that the pathological mutations in SOD1 are often gain of function, and yeast are particularly suitable for studying the molecular mechanism of action for each mutant allele [47]. In a Huntington's model, Htt103Q causes pronounced cytotoxicity in yeast, while the shorter, more soluble Htt25Q does not (Figure 2).

Many libraries exist for high-throughput genome-wide functional screening methods. The nonessential haploid deletion library encompasses 4850 strains, each lacking a single gene of interest [48]. In one screen, 52 gene deletions that enhance α-synuclein toxicity were isolated: about a third were involved in protein folding and cellular stress response, while another third were involved in vesicular trafficking and metabolism of lipids [49]. Conversely, the same library was screened for mutations that reduced Htt103Q toxicity [50]. Out of the major gene ontology families identified, about a fourth were involved in vesicular trafficking and vacuolar import and sorting, another one-fourth were involved in transcription and chromatin architecture, and another fourth were prions or prion-like proteins containing Q/N rich regions. These results imply a degree of overlap in management of toxic protein aggregates. Deletion of *BNA4* encoding KMO suppressed toxicity of Htt103Q in yeast and was found to be activated in patients and animal models of HD. KMO is the major route of tryptophan degradation in eukaryotes and metabolites of the pathway generate free radicals, suggestive of common pathologies in these disparate systems [51]. Beyond superficial life/death screens, these libraries can be exploited to drill down to specific features of protein aggregation diseases. Using the first exon of the *HD* gene fused to GFP, the Kmiec laboratory was able to visualize the Huntington's aggregates in living cells and screen for knockouts that affected aggregate formation [52]. Strains lacking the DNA mismatch repair genes *MSH2*, *MSH3* and *MLH1* exhibited reduced inclusion formation, leading to speculation that this pathway may aid in the reversal of polymerase slippage errors. In addition to gene knockout collections, random or ordered overexpression libraries have been used to identify gain-of-function modulators of protein aggregation phenotypes. In one screen, 77 overexpression suppressors of α-synuclein toxicity were found; the genes were involved in vesicular as well as metal ion transport, synthesis of osmolytes, phosphorylation of proteins, response to nitrosative as well as heat stress and biosynthesis/metabolism of trehalose (a chemical chaperone) [53]. The screen revealed the Rab GTPase Ypt1, the Tpo4 polyamine transporter and trehalose as suppressors of α-synuclein toxicity [46]. These findings correlate well with targeted investigation of physiological effects of α-synuclein expression in yeast, which localizes to plasma membranes and causes cytoplasmic vesicular accumulations similar to those seen in human PD [54]. Additionally vesicular trafficking is altered, levels of reactive oxygen species (ROS) increase and the heat shock response is stimulated. Consistently, Ypt1 was also found to associate with the α-synuclein accumulations [54]. **‘Humanized’ yeast** are also utilized in high-throughput screening of human protein libraries to discover novel, aggregation-prone proteins. One such study considered properties of TDP-43 and FUS and through bioinformatics analyzed 213 human proteins for those which had high probability for prion-like domains [55]. The proteins that resembled TDP-43 and FUS to the highest degree were then expressed in yeast to determine toxicity and aggregation capabilities [55]. Mutated forms of human TAF15, a general transcription factor, were found to be both toxic in yeast and to mis-localize to the cytoplasm of neurons in ALS patients, revealing a possible new pathogenic protein variant [55].

High-throughput screening of compounds that ameliorate protein aggregation disease phenotypes are also possible with yeast. Such screens require some fine-tuning because yeast has a thick cell wall and efficient membrane efflux pump system to rid the cell of foreign compounds [56]. Researchers have mutated genes involved in ergosterol biosynthesis (a cholesterol-like molecule required for membrane stability) or deleted members of the efflux pump system to overcome these caveats [56]. The screening of 16,000 compounds resulted in the discovery of nine small molecules that ameliorated Htt103Q growth inhibition [57]. One of the compounds, C2–8, was additionally shown to suppress aggregation of polyQ proteins within cell-cultured neurons from mice and prevent neurodegeneration in flies. In a screen of 5000 natural products an antioxidant found in green tea, epigallocatechin 3-gallate (EGCG), also prevented aggregation of Htt103Q [58]. In a 115,000 compound screen for supressors of α-synuclein toxicity, four structurally related compounds were found that reduced inclusion formation. The compounds were specific to α-synuclein toxicity as they did not suppress toxicity of Htt103Q [59]. Another study of 190,000 compounds established N-aryl benzimidazole as an effective agent to reduce and reverse α-synuclein toxicity in both yeast and in induced pluripotent stem cells [60,61]. The same group applied this compound in genetic screens and determined it caused an increase in vesicular trafficking through Rsp5(yeast)/Nedd4(mammalian) [60].

### Escherichia coli

Bacterial inclusion bodies, comprised of insoluble recombinant proteins, have certain amyloid-like qualities – they consist mainly of many copies of a single protein arranged in a cross beta sheet structure [62,63], have nucleation/seeding abilities [64] and bind Congo Red and thioflavin-T [64] – that allow them to be used as cheap and simple models of amyloidogenesis. One group designed a high-throughput screen where *E. coli* cells expressing green fluorescent protein (GFP) fused to the Alzheimer’s-related protein Aβ42 were grown in 96-well plates and screened against an extensive compound library for inhibitors of aggregation that kept the translational fusion soluble, allowing the GFP moiety time to fold properly and fluoresce [65]. The authors discovered the compound D737 prevented Aβ42 aggregation and increased the lifespan and locomotive skills in a *D. melanogaster* model of Alzheimer’s, demonstrating the utility of prokaryotic systems for investigations into protein misfolding diseases [66].

## Conclusion & future perspective

Model systems play an important role in understanding the etiology, impacts and physiological aspects of protein misfolding diseases. Cost, time and difficulty scale with organismal complexity, suggesting that investigators must balance multiple factors when evaluating different model systems for use. As detailed herein, models can be split into two major classes: those that allow investigation of disease pathology at the tissue, organ, and physiological levels, and those that permit probing the intricacies of protein misfolding diseases at the molecular level. Clearly rodents, and to some degree flies, fish and worms, fall into the first category, while the yeast and bacterial models are more appropriately considered ‘molecular’ systems. However, the artificial separation of these models into physiological and molecular groups may shortly be obsolete. As experimental obstacles are broken down and molecular tools mature in the larger animal systems, some of the advantages currently only available in yeast and bacteria may soon be realized in these models.

**Table 1.** **Comparison of model systems.**

**Organism**	**Generation time**	**Cost^†^/day**	**Protocol required?**	**Time to generate gene knockout^‡^**
Mouse	3 months	1$ (per cage)	Yes	3–4 months
Zebrafish	3–4 months	0.6$ (per tank)	Yes	3–4 months (3–4 weeks kd^§^)
Fruit fly	10 days	Negligible	No	1–3 months
Roundworm	4 days	Negligible	No	1–3 months (2–3 days kd^§^)
Baker's yeast	90 min	Negligible	No	3–4 days
Bacteria	20 min	Negligible	No	3–4 fays

^†^All costs shown in US dollars.

^‡^With extant mutant libraries, gene function can be immediately examined.

^§^kd: Transient knockdown.

**Key terms:**

**Wild-type:** Strain, cell line or animal expressing a functional, usually unaltered version of a given locus representative of a population.

**Knockdown:** Reducing expression of a gene or production of a gene product via molecular genetic techniques. Distinct from a knockout, wherein the gene product is completely missing. These two conditions frequently, but not always, result in identical outcomes.

**Humanized yeast:** Expressing one or more key human genes (usually in the form of cDNAs driven from a yeast promoter to allow for subsequent investigation of the human gene product in a heterologous context.

**Executive summary**

A variety of model systems are available to investigate pathology and mechanism of protein misfolding diseases.
The choice of model system must be appropriate for the pathology being investigated – for example, cognitive impairment in rodents, and molecular events/interactions in yeast.
Analyses of human protein misfolding disease-causing proteins in disparate systems reveal surprising functional conservation.
High-throughput screens for small molecule therapeutics in multiple systems allow complementary cross-platform investigation.

## References

[1] Van Dam D, De Deyn PP (2011). Animal models in the drug discovery pipeline for Alzheimer's disease. *Br. J. Pharmacol.*.

[2] Levin ED, Rose JE, Mcgurk SR, Butcher LL (1990). Characterization of the cognitive effects of combined muscarinic and nicotinic blockade. *Behav. Neural Biol.*.

[3] Yamada M, Chiba T, Sasabe J (2005). Implanted cannula-mediated repetitive administration of Aβ25–35 into the mouse cerebral ventricle effectively impairs spatial working memory. *Behav. Brain Res.*.

[4] Roberts TJ (2005). 3-Nitropropionic acid model of metabolic stress. *Methods Mol. Med.*.

[5] Sanberg PR, Calderon SF, Giordano M, Tew JM, Norman AB (1989). The quinolinic acid model of Huntington's disease: locomotor abnormalities. *Exp. Neurol.*.

[6] Ungerstedt U (1968). 6-Hydroxy-dopamine induced degeneration of central monoamine neurons. *Eur. J. Pharmacol.*.

[7] Prikhojan A, Brannan T, Yahr M (2000). Comparative effects of repeated administration of dopamine agonists on circling behavior in rats. *J. Neural Transmission*.

[8] Philipson O, Lord A, Gumucio A, O'callaghan P, Lannfelt L, Nilsson LN (2010). Animal models of amyloid-β-related pathologies in Alzheimer's disease. *FEBS J.*.

[9] Wirak D, Bayney R, Ramabhadran T (1992). Age-associated inclusions in normal and transgenic mouse brain. *Science*.

[10] Lamb BT, Sisodia SS, Lawler AM (1993). Introduction and expression of the 400 kilobase precursor amyloid protein gene in transgenic mice. *Nat. Genet.*.

[11] Irizarry MC, Soriano F, Mcnamara M (1997). Aβ deposition is associated with neurophil changes, but not with overt neuronal loss in the human amyloid precursor protein V717F (PDAPP) transgenic mouse. *J. Neurosci.*.

[12] Masliah E, Sisk A, Mallory M, Games D (2001). Neurofibrillary pathology in transgenic mice overexpressing V717F [beta]-amyloid precursor protein. *J. Neuropathol. Exp. Neurol.*.

[13] Ona VO, Li M, Vonsattel JPG (1999). Inhibition of caspase-1 slows disease progression in a mouse model of Huntington's disease. *Nature*.

[14] Chesselet M-F (2008). *In vivo* α-synuclein overexpression in rodents: a useful model of Parkinson's disease?. *Exp. Neurol.*.

[15] Li X, Patel JC, Wang J (2010). Enhanced striatal dopamine transmission and motor performance with LRRK2 overexpression in mice is eliminated by familial Parkinson's disease mutation G2019S. *J. Neurosci.*.

[16] Greeve I, Kretzschmar D, Tschäpe J-A (2004). Age-dependent neurodegeneration and Alzheimer-amyloid plaque formation in transgenic *Drosophila*. *J. Neurosci.*.

[17] Finelli A, Kelkar A, Song H-J, Yang H, Konsolaki M (2004). A model for studying Alzheimer's Aβ42-induced toxicity in *Drosophila melanogaster*. *Mol. Cell. Neurosci.*.

[18] Iijima K, Liu H-P, Chiang A-S, Hearn SA, Konsolaki M, Zhong Y (2004). Dissecting the pathological effects of human Aβ40 and Aβ42 in *Drosophila*: a potential model for Alzheimer's disease. *Proc. Natl Acad. Sci. USA*.

[19] Crowther D, Kinghorn K, Miranda E (2005). Intraneuronal Aβ, non-amyloid aggregates and neurodegeneration in a *Drosophila* model of Alzheimer's disease. *Neuroscience*.

[20] Whitworth AJ, Wes PD, Pallanck LJ (2006). *Drosophila* models pioneer a new approach to drug discovery for Parkinson's disease. *Drug Discov. Today*.

[21] Pendleton RG, Parvez F, Sayed M, Hillman R (2002). Effects of pharmacological agents upon a transgenic model of Parkinson's disease in *Drosophila melanogaster*. *J. Pharmacol. Exp. Ther.*.

[22] Lee W-CM, Yoshihara M, Littleton JT (2004). Cytoplasmic aggregates trap polyglutamine-containing proteins and block axonal transport in a *Drosophila* model of Huntington's disease. *Proc. Natl Acad. Sci. USA*.

[23] Pallos J, Bodai L, Lukacsovich T (2008). Inhibition of specific HDACs and sirtuins suppresses pathogenesis in a *Drosophila* model of Huntington's disease. *Hum. Mol. Genet.*.

[24] Agrawal N, Pallos J, Slepko N (2005). Identification of combinatorial drug regimens for treatment of Huntington's disease using *Drosophila*. *Proc. Natl Acad. Sci. USA*.

[25] Wolfgang WJ, Miller TW, Webster JM (2005). Suppression of Huntington's disease pathology in *Drosophila* by human single-chain Fv antibodies. *Proc. Natl Acad. Sci. USA*.

[26] Warrick JM, Chan HE, Gray-Board GL, Chai Y, Paulson HL, Bonini NM (1999). Suppression of polyglutamine-mediated neurodegeneration in *Drosophila* by the molecular chaperone HSP70. *Nat. Genet.*.

[27] Auluck PK, Chan HE, Trojanowski JQ, Lee VM-Y, Bonini NM (2002). Chaperone suppression of α-synuclein toxicity in a *Drosophila* model for Parkinson's disease. *Science*.

[28] Muchowski PJ, Wacker JL (2005). Modulation of neurodegeneration by molecular chaperones. *Nat. Rev. Neurosci.*.

[29] Jackson GR, Wiedau-Pazos M, Sang T-K (2002). Human wild-type tau interacts with wingless pathway components and produces neurofibrillary pathology in *Drosophila*. *Neuron*.

[30] Zhong H, Zou H, Semenov MV (2009). Characterization and development of novel small-molecules inhibiting GSK3 and activating Wnt signaling. *Mol. BioSyst.*.

[31] Zou H, Zhou L, Li Y (2009). Benzo-[ε]-isoindole-1, 3-diones as potential inhibitors of glycogen synthase kinase-3 (GSK-3). Synthesis, kinase inhibitory activity, zebrafish phenotype, and modeling of binding mode. *J. Med. Chem.*.

[32] Anichtchik OV, Kaslin J, Peitsaro N, Scheinin M, Panula P (2004). Neurochemical and behavioural changes in zebrafish *Danio rerio* after systemic administration of 6-hydroxydopamine and 1-methyl-4-phenyl-1, 2, 3, 6-tetrahydropyridine. *J. Neurochem.*.

[33] Bretaud S, Lee S, Guo S (2004). Sensitivity of zebrafish to environmental toxins implicated in Parkinson's disease. *Neurotoxicol. Teratol.*.

[34] Flinn L, Mortiboys H, Volkmann K, Köster RW, Ingham PW, Bandmann O (2009). Complex I deficiency and dopaminergic neuronal cell loss in parkin-deficient zebrafish (*Danio rerio*). *Brain*.

[35] Markaki M, Tavernarakis N (2010). Modeling human diseases in *Caenorhabditis elegans*. *Biotechnol. J.*.

[36] Haldimann P, Muriset M, Vigh L, Goloubinoff P (2011). The novel hydroxylamine derivative NG-094 suppresses polyglutamine protein toxicity in *Caenorhabditis elegans*. *J. Biol. Chem.*.

[37] Gohil VM, Offner N, Walker JA (2011). Meclizine is neuroprotective in models of Huntington's disease. *Hum. Mol. Genet.*.

[38] Daigle I, Li C (1993). apl-1, a *Caenorhabditis elegans* gene encoding a protein related to the human beta-amyloid protein precursor. *Proc. Natl Acad. Sci. USA*.

[39] Ewald CY, Raps DA, Li C (2012). APL-1, the Alzheimer's amyloid precursor protein in *Caenorhabditis elegans*, modulates multiple metabolic pathways throughout development. *Genetics*.

[40] Mccormick AV, Wheeler JM, Guthrie CR, Liachko NF, Kraemer BC (2013). Dopamine D2 receptor antagonism suppresses tau aggregation and neurotoxicity. *Biol. Psychiatry*.

[41] Munoz-Lobato F, Rodriguez-Palero MJ, Naranjo-Galindo FJ (2014). Protective role of DNJ-27/ERdj5 in *Caenorhabditis elegans* models of human neurodegenerative diseases. *Antioxid. Redox Signal.*.

[42] Li J, Huang KX, Le WD (2013). Establishing a novel *C. elegans* model to investigate the role of autophagy in amyotrophic lateral sclerosis. *Acta Pharmacol. Sin.*.

[43] Vaccaro A, Patten SA, Ciura S (2012). Methylene blue protects against TDP-43 and FUS neuronal toxicity in *C. elegans* and *D. rerio*. *PLoS ONE*.

[44] Outeiro TF, Muchowski PJ (2004). Molecular genetics approaches in yeast to study amyloid diseases. *J. Mol. Neurosci.*.

[45] Heinicke S, Livstone MS, Lu C (2007). The Princeton Protein Orthology Database (P-POD): a comparative genomics analysis tool for biologists. *PLoS ONE*.

[46] Pimentel C, Batista-Nascimento L, Rodrigues-Pousada C, Menezes RA (2012). Oxidative stress in Alzheimer's and Parkinson's diseases: insights from the yeast *Saccharomyces cerevisiae*. *Oxidat. Med. Cell. Longevity*.

[47] Bastow EL, Gourlay CW, Tuite MF (2011). Using yeast models to probe the molecular basis of amyotrophic lateral sclerosis. *Biochem. Soc. Trans.*.

[48] Winzeler EA, Shoemaker DD, Astromoff A (1999). Functional characterization of the *S. cerevisiae* genome by gene deletion and parallel analysis. *Science*.

[49] Willingham S, Outeiro TF, Devit MJ, Lindquist SL, Muchowski PJ (2003). Yeast genes that enhance the toxicity of a mutant huntingtin fragment or α-synuclein. *Science*.

[50] Giorgini F, Guidetti P, Nguyen Q, Bennett SC, Muchowski PJ (2005). A genomic screen in yeast implicates kynurenine 3-monooxygenase as a therapeutic target for Huntington disease. *Nat. Genet.*.

[51] Schwarcz R (2004). The kynurenine pathway of tryptophan degradation as a drug target. *Curr. Opin. Pharmacol.*.

[52] Hu Y, Liu L, Kmiec EB (2003). Reduction of Htt inclusion formation in strains of *Saccharomyces cerevisiae* deficient in certain DNA repair functions: a statistical analysis of phenotype. *Exp. Cell Res.*.

[53] Cooper AA, Gitler AD, Cashikar A (2006). α-synuclein blocks ER-Golgi traffic and Rab1 rescues neuron loss in Parkinson's models. *Science*.

[54] Soper JH, Roy S, Stieber A (2008). α-synuclein–induced aggregation of cytoplasmic vesicles in *Saccharomyces cerevisiae*. *Mol. Biol. Cell*.

[55] Couthouis J, Hart MP, Shorter J (2011). A yeast functional screen predicts new candidate ALS disease genes. *Proc. Natl Acad. Sci. USA*.

[56] Outeiro TF, Giorgini F (2006). Yeast as a drug discovery platform in Huntington's and Parkinson's diseases. *Biotechnol. J.*.

[57] Zhang X, Smith DL, Meriin AB (2005). A potent small molecule inhibits polyglutamine aggregation in Huntington's disease neurons and suppresses neurodegeneration *in vivo*. *Proc. Natl Acad. Sci. USA*.

[58] Ehrnhoefer DE, Duennwald M, Markovic P (2006). Green tea (−)-epigallocatechin-gallate modulates early events in huntingtin misfolding and reduces toxicity in Huntington's disease models. *Hum. Mol. Genet.*.

[59] Khurana V, Lindquist S (2010). Modelling neurodegeneration in *Saccharomyces cerevisiae*: why cook with baker's yeast?. *Nat. Rev Neurosci*.

[60] Tardiff DF, Jui NT, Khurana V (2013). Yeast reveal a “druggable” Rsp5/Nedd4 network that ameliorates α-synuclein toxicity in neurons. *Science*.

[61] Chung CY, Khurana V, Auluck PK (2013). Identification and rescue of α-synuclein toxicity in Parkinson patient-derived neurons. *Science*.

[62] Carrio M, Corchero J, Villaverde A (1998). Dynamics of *in vivo* protein aggregation: building inclusion bodies in recombinant bacteria. *FEMS Microbiol. Lett.*.

[63] Morell M, Bravo R, Espargaró A (2008). Inclusion bodies: specificity in their aggregation process and amyloid-like structure. *Biochim. Biophys. Acta Mol. Cell Res.*.

[64] Carrió M, González-Montalbán N, Vera A, Villaverde A, Ventura S (2005). Amyloid-like properties of bacterial inclusion bodies. *J. Mol. Biol.*.

[65] Kim W, Kim Y, Min J, Kim DJ, Chang Y-T, Hecht MH (2006). A high-throughput screen for compounds that inhibit aggregation of the Alzheimer's peptide. *ACS Chem. Biol.*.

[66] Mckoy AF, Chen J, Schupbach T, Hecht MH (2012). A novel inhibitor of amyloid β (Aβ) peptide aggregation form high throughput screening to efficacy in an animal model of Alzheimer disease. *J. Biol. Chem.*.

